# Supplementary material for: MRI differentiation of usual type endocervical adenocarcinoma and cervical squamous cell carcinoma
Source: PLoS One. 2026 Feb 27;21(2):e0343764. doi: 10.1371/journal.pone.0343764 (PMC12948134; doi:10.1371/journal.pone.0343764)
Supplement: Attachment — Submitted filename: Revision letter 31.12.2025 Ebru_Hasbay.docx [file pone.0343764.s003.docx]

**Manuscript ID:** PONE-D-25-41538

**Title:** MRI Differentiation of Usual Type Endocervical Adenocarcinoma and Cervical Squamous Cell Carcinoma

Dear Dr. Woloschak and the Editorial Team,

We sincerely thank the Section Editor and both reviewers for their careful evaluation of our manuscript and for the constructive comments that substantially improved the rigor, clarity, and interpretability of our work. We have revised the manuscript extensively in response to all points raised.

**Reviewer #1**

**1) “ADC cutoff: Which criterion was used? Consider Youden index.”**

**Response:** We agree. We now explicitly state that the optimal ADC cut-off was determined using the **Youden index (J = sensitivity + specificity − 1)**. The Youden-derived threshold was **0.837 × 10⁻³ mm²/s**, yielding **65.4% sensitivity** and **62.0% specificity** for identifying UEA.
**Manuscript changes:** Added/clarified in **Statistical analysis** and **ROC Analysis** sections.

**2) “Retrospective study: multivariable analysis is essential; univariate ADC conclusion is not strong enough.”**

**Response:** We agree and added a **multivariable logistic regression** model to determine independent predictors of UEA versus SCC. In the final model, **mean ADC value** and **tumor growth pattern** were independent predictors of UEA.
**Manuscript changes:** Added to **Statistical analysis** and **Results—Multivariable Analysis**; Discussion updated to emphasize multiparametric interpretation.

**3) “Only 26 UEA and 50 SCC: include no more than 2–3 predictors; clarify overfitting.”**

**Response:** We agree. To minimize overfitting risk, we **restricted the number of predictors** in the multivariable model. We also explicitly acknowledge overfitting risk and limited sample size in the **Limitations** section.
**Manuscript changes:** Clarified in **Statistical analysis** (model restriction rationale) and expanded in **Limitations**.

**4) “Compare predictive power between DWI and ADC using AUC with 95% CIs; compare AUCs statistically (DeLong). At minimum report 95% CI.”**

**Response:** Implemented. We now report **AUC with 95% CI** for both ADC and DWI. We also performed **DeLong’s test** for correlated ROC curves. DWI showed numerically higher AUC, but the difference was **not statistically significant** (**ΔAUC = 0.055; p = 0.592**).
**Manuscript changes:** Added to **Statistical analysis** and **Results—ROC Analysis**.

**5) “Table 1: p < 0.1 variables (e.g., FIGO) can be considered in multivariable analysis.”**

**Response:** Thank you. Consistent with this recommendation, we state that variables with **p < 0.10** in univariable analyses and/or clinically relevant variables were considered for entry. Given the limited sample size, we prioritized a parsimonious model to reduce overfitting.
**Manuscript changes:** Clarified variable entry rule and parsimony rationale in **Statistical analysis**.

**6) “Figure 2 ROC: ADC not a very good predictor. Run multivariable models for better combination.”**

**Response:** We agree. We tempered our interpretation of ADC’s diagnostic strength and emphasized that ADC alone has **moderate discrimination**. We added a **multivariable model** and revised the Discussion/Conclusion to highlight that best performance comes from a **multiparametric approach**, not a single predictor.
**Manuscript changes:** Revised in **Results—ROC Analysis**, **Results—Multivariable Analysis**, **Discussion**, and **Conclusion**.

**Reviewer #2**

**Abstract**

**1) “Abstract overstates diagnostic value; does not mention low specificity (0.44). Include AUC/CI; summarize negative findings.”**

**Response:** We agree and revised the Abstract to provide AUC with 95% CI and a more balanced interpretation. In the revised analysis, the ADC cut-off derived by the Youden index yielded **specificity 62.0%** (not 0.44). We also reduced overstatement and noted that ADC provides **moderate** discrimination.
**Manuscript changes:** Revised **Abstract—Results/Conclusion**.

**Introduction**

**2) “UEA should be clearly defined.”**

**Response:** We added a clear definition of UEA and its place within HPV-associated endocervical adenocarcinomas, and clarified why focusing specifically on UEA is relevant.
**Manuscript changes:** Revised **Introduction**, first paragraphs.

**3) “Rephrase: ‘UEA is more aggressive’ and ‘prognosis similar to SCC’—literature inconsistent.”**

**Response:** We revised the wording to reflect that the literature is **not fully consistent**, citing studies showing both more aggressive behavior for adenocarcinoma as a group and similar prognosis for UEA in some cohorts.
**Manuscript changes:** Revised **Introduction** (prognosis statements).

**4) “MRI differentiation has been studied for AC vs SCC; explain why UEA vs SCC is clinically meaningful.”**

**Response:** We clarified the clinical rationale: UEA is the most common adenocarcinoma subtype and may show distinctive growth patterns and internal architecture; subtype-level identification may improve preoperative characterization and surgical/radiotherapy planning.
**Manuscript changes:** Revised **Introduction** (study rationale paragraph).

**5) “End introduction with clear hypothesis.”**

**Response:** We added a clear study aim/hypothesis stating that quantitative diffusion metrics (ADC) and qualitative growth pattern may help differentiate UEA from SCC.
**Manuscript changes:** Added final sentence of **Introduction**.

**Methods**

**6) “Describe whether cases were consecutive or randomly selected.”**

**Response:** We clarified that **all consecutive eligible patients** within the study period who met inclusion criteria and had preoperative MRI were included.
**Manuscript changes:** Revised **Methods—Patients**.

**7) “Provide scan parameters for all sequences.”**

**Response:** We expanded the MRI protocol description and provided acquisition details (scanner types, planes, key parameters, DWI b-values, ADC generation).
**Manuscript changes:** Expanded **Methods—MRI protocol**.

**8) “Inter-scanner variability; ADC available only for 18/26 UEA; describe missing data handling.”**

**Response:** In the revised manuscript, **ADC measurements were available for all included patients**, and we state this explicitly. We also acknowledged potential inter-scanner variability as a limitation due to multiple 1.5T systems and long study period.
**Manuscript changes:** Clarified in **Methods—MRI protocol** (“ADC measurements were successfully obtained for all patients included in the final analysis.”) and expanded **Limitations** regarding inter-scanner variability.

**9) “Consensus discrepancies: show them in results.”**

**Response:** We addressed this by adding comprehensive **interobserver agreement** results: ICCs for quantitative variables and Cohen’s κ for qualitative features, demonstrating excellent agreement; discrepancies were resolved by consensus.
**Manuscript changes:** Added/expanded **Results—Interobserver Agreement** and **Table 5**.

**10) “Provide ROI details: size/number/averaging across slices.”**

**Response:** We added detailed ROI methodology: circular ROI placed as large as possible on the largest tumor slice, avoiding hemorrhage/necrosis, referencing other sequences; **≥3 measurements** were obtained and averaged.
**Manuscript changes:** Expanded **Methods—Image analysis**.

**11) “Multivariate logistic regression needed; consider confounders (FIGO, menopausal status, scanner).”**

**Response:** We added multivariable logistic regression. Due to sample size constraints and overfitting risk, we used a parsimonious model. Potential confounding by FIGO stage, menopausal status, and scanner variability is now explicitly discussed as a limitation and rationale for future prospective validation.
**Manuscript changes:** Added **Multivariable analysis** and expanded **Limitations**.

**12) “Not clear how SI was required.”**

**Response:** We clarified the role of SI in the analysis: SI was assessed qualitatively on T1WI/T2WI/DWI/CE-T1WI; additionally, tumor-to-muscle SIRs were calculated for quantitative comparison.
**Manuscript changes:** Clarified in **Methods—Image analysis**.

**Results**

**13) “ADC misreported (0.825 ± 102.07 etc.). Correct; consider median/IQR if non-normal; provide CI/effect sizes.”**

**Response:** We corrected ADC reporting and now present values as **mean ± SD** with appropriate units. Normality was assessed by Shapiro–Wilk, and tests were chosen accordingly. We additionally report **mean difference with 95% CI** and **effect size (Hedges’ g)** for ADC.
**Manuscript changes:** Revised **Results—Quantitative MRI Evaluation**.

**14) “Marginal differences (FIGO p=0.073, tumor size p=0.090, CE-T1WI SIR p=0.055): describe/discuss and compare with prior studies; describe non-significant findings.”**

**Response:** We expanded the Results text to explicitly report these borderline findings and added discussion emphasizing cautious interpretation given multiple comparisons and limited power. We also summarized that remaining quantitative/qualitative features did not differ significantly between groups.
**Manuscript changes:** Revised **Results** and expanded **Discussion**.

**15) “Specificity 0.44 indicates modest performance; report and interpret cautiously.”**

**Response:** We agree. In the revised ROC analysis, the Youden-derived ADC threshold yielded **specificity 62.0%** (and sensitivity 65.4%), with **AUC 0.659**, indicating **moderate** discrimination. We revised interpretation to be cautious and present ADC as supportive rather than definitive.
**Manuscript changes:** Revised **Results—ROC Analysis** and **Discussion**.

**16) “No P values for Table 4.”**

**Response:** Thank you. We added the missing p values for Table 4 and ensured consistency between text and tables.
**Manuscript changes:** Corrected **Table 4**.

**Discussion**

**17) “Compare with prior MRI studies on adenocarcinomas and SCC.”**

**Response:** We expanded the Discussion to compare our findings with previously published MRI studies addressing cervical adenocarcinoma subtypes, SCC, diffusion metrics, growth patterns, and cystic components, and we added relevant citations.
**Manuscript changes:** Expanded **Discussion** with additional references.

**18) “Modest AUC/specificity weaken diagnostic meaningfulness; temper claims.”**

**Response:** Implemented. We revised Discussion and Conclusion to avoid overstatement, emphasizing **moderate** diagnostic performance and the need for **multiparametric assessment** and validation.
**Manuscript changes:** Revised **Discussion** and **Conclusion**.

**19) “Speculation about cystic components/cellularity without histopath correlation.”**

**Response:** We agree and revised the wording to indicate these are **plausible explanations** rather than proven mechanisms, and we explicitly note the absence of quantitative histopathologic correlation as a limitation.
**Manuscript changes:** Revised **Discussion** and added to **Limitations**.

**Limitations**

**20) “Mention missing ADC, inter-scanner variability; lack of validation/cross-validation as key weakness; suggest future multicenter/prospective.”**

**Response:** We expanded the Limitations accordingly. In the revised dataset, ADC values were available for all included patients; nevertheless, we explicitly discuss **inter-scanner/protocol variability**, **single-center retrospective design**, and **lack of external or internal validation** as major limitations, and we recommend **prospective multicenter validation** with standardized protocols.
**Manuscript changes:** Expanded **Limitations** section.

**Conclusion**

**21) “Conclusion overstates clinical applicability; rephrase.”**

**Response:** We revised the Conclusion to a more cautious tone, stating that findings are supportive and require validation, and that ADC alone is insufficient as a standalone discriminator.
**Manuscript changes:** Revised **Conclusion**.

**Other / Editorial points**

**22) “Provide full name at first use for abbreviations.”**

**Response:** Implemented throughout the manuscript.
**Manuscript changes:** Checked across **Abstract/Introduction/Methods/Results**.

**23) “Missing punctuation: ‘rate in the diagnosis… Thus, MRI…’”**

**Response:** Corrected and edited for clarity.
**Manuscript changes:** Revised **Introduction**.

**24) “‘IB–4B’ → ‘IB–ⅣB’.”**

**Response:** Corrected the FIGO stage notation.
**Manuscript changes:** Revised **Methods—Patients**.

**25) “Data availability inconsistency (‘fully available without restriction’ vs ‘reasonable request’).”**

**Response:** We aligned the Data Availability Statement to a single, consistent wording: data are **available from the corresponding author upon reasonable request.**

**Manuscript changes:** Revised **Title page / Declarations—Data Availability**.

**26) “Grammatical/typographical inconsistencies.”**

**Response:** We performed a thorough language edit to correct grammar, punctuation, and consistency in units/formatting.
**Manuscript changes:** Applied throughout.

**27) “Peer review history option: what does this mean?”**

**Response:** We understand that if authors opt in, PLOS ONE may publish the peer review history (decision letters, reviewer comments, and author responses/attachments). This is an editorial option and does not change the scientific content of the manuscript. (No manuscript text change required.)
