# Supplementary material for: MRI differentiation of usual type endocervical adenocarcinoma and cervical squamous cell carcinoma
Source: PLoS One. 2026 Feb 27;21(2):e0343764. doi: 10.1371/journal.pone.0343764 (PMC12948134; doi:10.1371/journal.pone.0343764)
Supplement: Attachment — Submitted filename: Second revision letter Ebru_Hasbay 12.01.2026.DOCX [file pone.0343764.s004.DOCX]

Response to Reviewer #1 (Xiaoyue Ma)

Comment 1: The authors noted in the results that both AUC and sensitivity/specificity determined by the Youden index point to the very limited discriminative ability of the model. Meanwhile, in multivariate analysis, mean ADC values and tumor growth pattern were found significant. However, the OR for mean ADC values is only 1.006, which is significant but the clinical significance of this association is debatable.

Response: We agree with the reviewer that while the mean ADC value is a statistically significant independent predictor (p = 0.009), its odds ratio (OR = 1.006) indicates a small clinical effect size per unit increase. We have addressed this in the Discussion section to clarify that ADC should be viewed as a supportive biomarker rather than a definitive standalone diagnostic tool.

Revision in Manuscript (Discussion): > "Although mean ADC was an independent predictor in our multivariable model, its odds ratio (1.006) suggests a small clinical effect size per unit change. Consequently, it should be considered a supportive parameter to be integrated with other MRI features within a multiparametric assessment, rather than a standalone definitive diagnostic tool."

Comment 2: For variable selections in multivariable logistic regression, we cannot only rely on the P-value from univariate analysis to decide which variables to keep, as there might be high correlation between certain predictors. Will the authors consider the forward/backward selection method to decide the variables to be kept in the model (P<0.10 in univariate analysis)?

Response: We thank the reviewer for this excellent methodological suggestion. Following this recommendation, we have re-analyzed our data using a backward stepwise selection method (based on Likelihood Ratio) for the multivariable logistic regression. All variables with p < 0.10 in the univariate analysis (tumor size, growth pattern, and mean ADC) were initially included. The stepwise procedure confirmed that mean ADC and tumor growth pattern were the most robust independent predictors for UEA. We have updated the Methods and Results sections to reflect this change in methodology.

Revision in Manuscript (Methods): > "To identify independent predictors of UEA, a multivariable logistic regression analysis was performed using a backward stepwise selection method (Likelihood Ratio). Variables that reached a p-value <0.10 in the univariate analysis were initially included in the model."

Revision in Manuscript (Results): > "The multivariable logistic regression model, refined through backward stepwise selection, remained statistically significant " (Omnibus χ² = 13.35, df = 2, p = 0.001) with adequate calibration. "
